# Assessment of distribution and evolution of Mechanical dyssynchrony in a porcine model of myocardial infarction by cardiovascular magnetic resonance

**DOI:** 10.1186/1532-429X-14-1

**Published:** 2012-01-06

**Authors:** Khaled Z Abd-Elmoniem, Miguel Santaularia Tomas, Tetsuo Sasano, Sahar Soleimanifard, Evert-Jan P Vonken, Amr Youssef, Harsh Agarwal, Veronica L Dimaano, Hugh Calkins, Matthias Stuber, Jerry L Prince, Theodore P Abraham, M Roselle Abraham

**Affiliations:** 1 Biomedical and Metabolic Imaging Branch, National Institute of Diabetes and Digestive and Kidney Diseases, NIH, Bethesda, MD, USA; 2 Department of Medicine, Division of Cardiology, Johns Hopkins University, Baltimore, MD, USA; 3 Department of Medicine, Division of Cardiology, Hospital Español de Mexico, Distrito Federal, Mexico; 4 Department of Electrical and Computer Engineering, Johns Hopkins University, Baltimore, MD, USA; 5 Department of Radiology, Division of Magnetic Resonance Research, Johns Hopkins University, Baltimore, MD, USA

## Abstract

**Background:**

We sought to investigate the relationship between infarct and dyssynchrony post- myocardial infarct (MI), in a porcine model. Mechanical dyssynchrony post-MI is associated with left ventricular (LV) remodeling and increased mortality.

**Methods:**

Cine, gadolinium-contrast, and tagged cardiovascular magnetic resonance (CMR) were performed pre-MI, 9 ± 2 days (early post-MI), and 33 ± 10 days (late post-MI) post-MI in 6 pigs to characterize cardiac morphology, location and extent of MI, and regional mechanics. LV mechanics were assessed by circumferential strain (eC). Electro-anatomic mapping (EAM) was performed within 24 hrs of CMR and prior to sacrifice.

**Results:**

Mean infarct size was 21 ± 4% of LV volume with evidence of post-MI remodeling. Global eC significantly decreased post MI (-27 ± 1.6% vs. -18 ± 2.5% (early) and -17 ± 2.7% (late), p < 0.0001) with no significant change in peri-MI and MI segments between early and late time-points. Time to peak strain (TTP) was significantly longer in MI, compared to normal and peri-MI segments, both early (440 ± 40 ms vs. 329 ± 40 ms and 332 ± 36 ms, respectively; p = 0.0002) and late post-MI (442 ± 63 ms vs. 321 ± 40 ms and 355 ± 61 ms, respectively; p = 0.012). The standard deviation of TTP in 16 segments (SD16) significantly increased post-MI: 28 ± 7 ms to 50 ± 10 ms (early, p = 0.012) to 54 ± 19 ms (late, p = 0.004), with no change between early and late post-MI time-points (p = 0.56). TTP was not related to reduction of segmental contractility. EAM revealed late electrical activation and greatly diminished conduction velocity in the infarct (5.7 ± 2.4 cm/s), when compared to peri-infarct (18.7 ± 10.3 cm/s) and remote myocardium (39 ± 20.5 cm/s).

**Conclusions:**

Mechanical dyssynchrony occurs early after MI and is the result of delayed electrical and mechanical activation in the infarct.

## Background

Cardiac resynchronization therapy (CRT) relieves symptoms, induces reverse remodeling, reduces heart failure hospitalizations, and improves survival in symptomatic heart failure patients with left ventricular systolic dysfunction and conduction abnormality [1-4]. However, CRT is plagued by a high non-responder rate of ~30% [5]. Differences between ischemic and non-ischemic cardiomyopathy (CM) have generally been overlooked in the early discussions on CRT. However, there are several clinically relevant differences between these two groups. Patients with ischemic CM are more likely to have higher scar burden and be non-responders with lower rates of symptomatic improvement, reverse remodeling, and increment in left ventricular (LV) function [6]. Furthermore, recent data suggest that dyssynchrony in the setting of an acute myocardial infarction (MI) portends a poor prognosis and predicts LV remodeling [7-11].

These clinical observations intensify the need for a more detailed investigation of regional mechanics following MI. Such knowledge may potentially help explain the differences in response to CRT between ischemic and non-ischemic CM and could potentially assist in developing novel treatment strategies for dyssynchrony in the post-MI setting. To date, most physiologic, mechanical, cellular, and molecular data concerning CRT have been generated in a well-validated tachy-pacing model of heart failure [12-14], but a similar thorough evaluation has been lacking in the setting of ischemic CM.

Cardiovascular magnetic resonance (CMR) is an attractive modality to study dyssynchrony because it permits simultaneous assessment of anatomy, scar burden, and myocardial deformation at high spatial and temporal resolution in experimental (small and large animal) models and patients. In this study, we used a well-characterized porcine model of MI [15] to determine the patterns and evolution of regional mechanics and dyssynchrony. Using CMR and electro-anatomic mapping, we found that mechanical dyssynchrony occurs early after acute MI and is due to delayed mechanical and electrical activation of the infarcted region. Hence, electrical CRT alone would not be expected to provide any benefit in this setting.

## Methods

### Animal Model

Our experimental protocol was approved by the Institutional Animal Care and Use Committee.

### Creation of myocardial infarction

Ten young farm pigs weighing 25 to 35 kg were subjected to percutaneous occlusion of the left anterior descending artery as was previously described [15]. Animals were imaged pre-MI, 9 ± 2d (early post-MI) and 33 ± 10d (late post-MI) post-MI. Please see supplemental data section for details.

### Cardiac Magnetic Resonance

Studies were performed using a clinical Philips 3.0T Achieva MR scanner (Philips Medical Systems, Best, NL) equipped with a six-channel cardiac phased array surface coil. Animals were mechanically ventilated, anaesthetized, and paralyzed for the duration of imaging. Cine, contrast-enhanced and tagged images [16] were obtained. Please see the additional file 1 for the surgical procedure, imaging protocols, and image analysis details. Additional file 2 demonstrates the notations used in this study for different myocardial segments.

### Image Analysis

LV mass and volumes, late gadolinium enhancement (LGE), and circumferential strain (eC) were measured using a custom-built software tool developed using Matlab^® ^ver. 7.6 (Mathworks, Natick, MA). Please see additional file 1 for details.

Dyssynchrony index was calculated as the standard deviation of time to peak eC (TTP) for 16 segments per animal, where time to peak strain was measured from the QRS complex. This index has been previously used to assess dyssynchrony in experimental and clinical studies [12,13,17-20]. The primary comparisons were made between infarct, peri-infarct, and normal segments at three time-points, namely, pre-MI (baseline), early (7-11 days), and late (30-40 days) post-MI.

### Electroanatomical Mapping (EAM)

EAM was performed using the CARTO system (CARTO XP, Biosense-Webster Inc.) in all 6 pigs within 24 hrs of CMR and prior to sacrifice. Please see additional file 1 for details.

### Statistical Analysis

All data were analyzed using JMP version 9 software (SAS Inc, Cary NC). Continuous variables are expressed as mean ± SD. Differences between regions and time points were tested using two way repeated measures ANOVA and a paired t test. A p value < 0.05 was considered significant. Inter-observer reproducibility was tested in 63 randomly selected segments, at the early and late post-MI time-points.

## Results

Of 10 animals, 4 died due to incessant ventricular fibrillation (1 intra-operatively during balloon occlusion and 3 in the immediate post operative period) leaving 6 animals for the study. We analyzed a total of 96 segments at baseline and 95 normal, 57 peri-MI and 33 MI segments at the early and late post-MI time-points. There was no significant difference in heart rate between the baseline, early and late post-MI time-points (Table 1).

**Table 1:** Baseline and Post-Infarct Remodeling

	Baseline (n = 6)	Early (n = 6)	Late (n = 6)	p-value
**Days after MI**	-	10 ± 2	34 ± 10	NA
**Heart rate (bpm)**	89 ± 8	74 ± 9	77 ± 15	NS
**LVEF (%)**	52 ± 5	44 ± 7	45 ± 4	< 0.01
**LVEDD (mm)**	38 ± 2	40 ± 3	41 ± 3	NS
**RVEDD (mm)**	24 ± 2	26 ± 2	27 ± 2	NS
**LVEDV (mL)**	42 ± 7	56 ± 10	65 ± 9	< 0.01
**LVESV (mL)**	20 ± 5	31 ± 7	36 ± 12	< 0.01
**MI segment thickness (mm)**	-	6 ± 1	5 ± 1	< 0.01
**Normal segment thickness (mm)**	9 ± 1	10 ± 0	12 ± 2	NS
**Late gadolinium enhancement %**	-	21 ± 4	20 ± 6	NS

EF = ejection fraction; EDD = end diastolic dimension; EDV and ESV = end-diastolic and end-systolic volumes, respectively. All p-values relate to early and late versus baseline except where baseline values are absent.

Regional myocardial contractility was assessed by segmental eC and eR. We were able to obtain adequate quality of eC and eR tracings from 283 (98%) and 172 (60%) of 288 total segments, respectively, at all 3 time-points. Since the yield was low and signal quality was unreliable for eR, we restricted all analysis to segmental eC only.

### Characterization of the infarct

**Figure 1:**
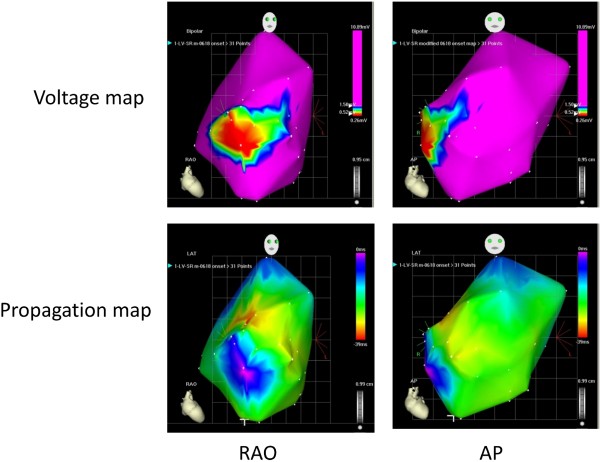
**Representative voltage and activation maps from 1 animal using the CARTO system (CARTO XP, Biosense-Webster Inc**.). The infarcted region was characterized by low voltage (< 0.5 mV) and delayed activation.

Cine and LGE images were used to characterize the infarct. Infarction was located in the apical to mid-anterior wall and anterior septum. All MI segments had LGE of ≥ 90% of myocardial wall thickness and were transmural. Mean infarct volume was 21 ± 4% and 20 ± 6% of the LV myocardial volume at early and late time-points, respectively (p = NS; Table 1, Figures 1, 3).

Post-infarct remodeling in the wall and ventricular chamber was confirmed by the following findings: 1) wall thickness was lower in the MI segments compared to the normal segments at the early time-point, and further decreased at the late post-MI time-point (Table 1). 2) end-diastolic and end-systolic LV volumes increased and EF decreased from baseline to early/late post-MI time-points (Table 1). However, left and right ventricular diameters, calculated at the mid-ventricular level did not change significantly between pre-MI, early and late post-MI time-points, which is probably because of the apical location of the infarct.

**Figure 2:**
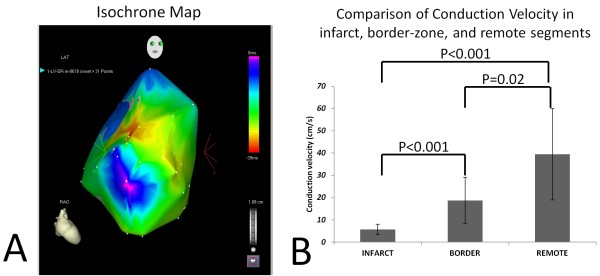
**A. Representative isochrone map (corresponding to voltage/activation maps in Figure 1) from 1 animal using the CARTO system (CARTO XP, Biosense-Webster Inc.) reveals crowding of isochrones indicative of greatly reduced conduction velocity in the infarct**. B. Summary of conduction of velocities in the infarct, border-zone and remote myocardium derived from all pigs (n = 6), using the CARTO system. Conduction velocity was very low in the infarct and intermediate in the infarct border-zone region, when compared to remote myocardium.

In the late post-MI period, EKG revealed normal sinus rhythm without evidence of bundle branch block. Electro-anatomic mapping late post-MI revealed low voltage and fractionated, low-amplitude electrograms in the anterior septum and mid/apical anterior wall during sinus rhythm in all animals, suggestive of scar (Figure 1). We found earliest ventricular activation in the anterior septum and septal to lateral activation in all animals; the infarct was the latest activated region (Figure 1). Figure 2A illustrates the isochrone map corresponding to voltage activation maps in Figure 1 demonstrating reduced conduction velocity in the infarct region. Conduction velocity was very low in the infarct (5.7 ± 2.4 cm/s) and intermediate in the infarct border-zone region (18.7 ± 10.3 cm/s; p < 0.001 infarct vs. IBZ), when compared to remote myocardium (39 ± 20.5 cm/s; p < 0.001 infarct vs remote; p = 0.02 for IBZ vs. remote)- Figure 2B.

**Figure 3:**
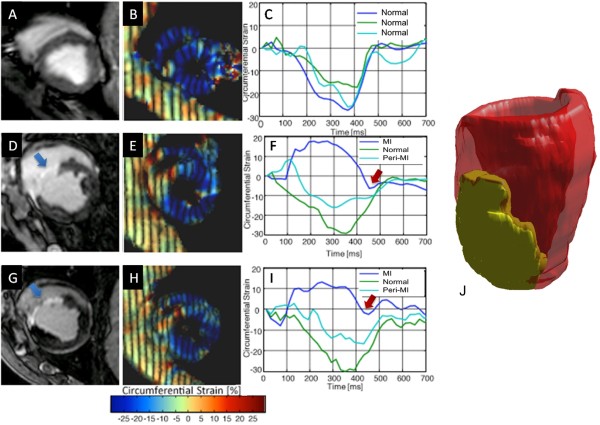
**Representative left ventricular short-axis CMR images illustrating absence of LGE pre-infarct**. (A) with normal color-coded strain display (B) and strain tracings showing synchronized mechanical activity with all segments peaking near simultaneously (C); Early post-MI images show LGE in the septum (D, arrow) with low strain in the septum (E) and strain traces showing mechanical dyssynchrony from early stretching (dyskinesis) and late hypokinesis of the infarcted segment (F). Late post- MI images show persistent LGE in the septum (G, arrow) with low strain in the septum (H) and strain traces showing mechanical dyssynchrony from early stretching (dyskinesis) and late hypokinesis of the infarcted segment (I). Representative 3-dimensional reconstruction of the area of delayed enhancement illustrating the extent of infarction (J).

### Global and Regional Mechanics

We compared the evolution of eC in MI, peri-MI and normal segments, separately and globally as an average of all 16 segments at each time point. Figure 3 illustrates LGE pattern, color-coded strain maps on zHARP tagged images [16,21], and strain tracings during one cardiac cycle from one animal. Synchronized mechanical activity with all segments peaking near simultaneously is evident pre-MI (Figure 3C). In the first week post-MI, low strain (Figure 3D), mechanical dyssynchrony, early stretching (dyskinesis) and late hypokinesis, are evident in the strain traces (Figure 3E) in the infarct segment. Similar findings were observed late post-MI (Figure 3H and 3I). Global peak eC (average eC for 16 segments) decreased significantly from pre-MI to early and late post MI (-27 ± 1.6% vs -18 ± 2.5% and -17 ± 2.7%, respectively, p value < 0.0001). There was no significant decrease in peak eC between early and late post MI (-18 ± 2.5% vs. -17 ± 2.7%, respectively, p = 0.55; Table 2).

**Table 2:** Global Mechanics

	Baseline	Early	Late	p value
Global peak eC	-27 ± 2	-18 ± 3	-17 ± 3	< 0.0001*
SD16 segments (dyssynchrony index)	28 ± 7	50 ± 10	54 ± 19	0.0079*
**Normal**				
Peak eC Normal	-27 ± 2	-24 ± 4	-21 ± 2	0.0032*
TTP Normal	300 ± 46	329 ± 40	321 ± 40	0.47
**Peri-MI**				
Peak eC Peri-MI	-27 ± 2	-18 ± 2	-17 ± 1	< 0.0001*
TTP Peri-MI	300 ± 46	332 ± 36	355 ± 61	0.18
**MI**				
Peak eC MI	-27 ± 2	-6 ± 2	-8 ± 2	< 0.0001*
TTP MI	300 ± 46	440 ± 40	442 ± 63	0.0002*

* No significant difference between early and late post-MI. SD16 = mean standard deviation of time to peak strain in 16 segments; MI = myocardial infarction

#### Analysis by time point

**Figure 4:**
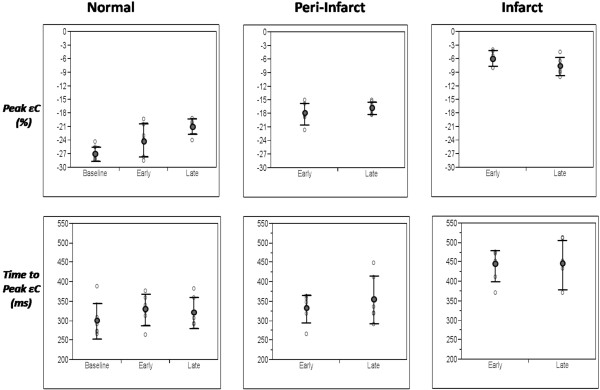
**Scatter plots illustrating peak strain (eC) and time to peak strain (TTP) at baseline, early and late post-MI time-points for normal, peri-MI and MI segments**. MI segments demonstrate low strain and delayed mechanical activation at early and late post-MI time-points. Peri-MI segments demonstrate reduction in eC with no significant delay in TTP at both time-points.

ANOVA displayed variation of Peak eC between three time points (p < 0.0001). Peak eC in *normal segments *decreased non-significantly from pre-MI to early post-MI (-27 ± 1.6% to -24 ± 3.7%, p = 0.5) but decreased significantly between pre-MI and late post-MI (-27 ± 1.6% to -21 ± 1.7%, p = 0.0008). There was a non-significant trend towards decrease in peak eC between early and late post MI (-24 ± 3.7% to -21 ± 1.7%, p = 0.06). However, despite these statistical differences, eC values at all 3 time-points were within normal range (Figure 4).

Peak eC significantly decreased from pre-MI to early and late post MI in *peri-MI *(-27 ± 1.6%, -18 ± 2.4%, -17 ± 1.5%, p = 0.0001) and *MI segments *(-27 ± 1.6%, -5.9 ± 1.7%, -7.7 ± 1.8%, p = 0.0001). There was no significant decrease in peak eC in peri-MI and MI segments between early and late post MI (-18 ± 2.4%, -17 ± 1.5%, p = 0.24 and -5.9 ± 1.7%, -7.7 ± 1.8%, p = 0.11; Table 2, Figure 4).

#### Analysis by region

ANOVA showed variation of Peak eC between MI, peri-MI, and normal regions (p = 0.0003). Peak segmental eC decreased significantly in normal, peri-MI, and MI segments between early (-24 ± 3.7, -18 ± 2.4 and -6 ± 1.7%, respectively; p < 0.001) and late MI time points (-21 ± 1.7, -17 ± 1.5 and -8 ± 2%, respectively; p < 0.001; Table 3, Figure 4).

**Table 3:** Evolution of Regional Mechanics

	Normal	Peri-Infarct	Infarct	p value
**Peak eC Early**	-24 ± 4	-18 ± 2	-6 ± 2	< 0.0001
**TTP Early**	329 ± 40	332 ± 36	440 ± 40	0.0002*
**Peak eC Late**	-21 ± 2	-17 ± 1	-8 ± 2	< 0.0001
**TTP Late**	321 ± 40	355 ± 61	442 ± 63	0.0054*

* No significant difference between normal and peri-MI

### Mechanical Dyssynchrony

#### Analysis by region

TTP between MI, peri-MI and normal regions varied significantly as assessed by ANOVA (p < 0.0001). The mean TTP for all 16 segments was 300 ± 28 ms at baseline. There were no statistically significant differences in TTP between normal and peri-MI segments at the early (329 ± 40 ms and 332 ± 36 ms; p = 0.92) and late post-MI (321 ± 40 ms and 355 ± 61 ms; p = 0.31) time-points. In contrast, TTP was significantly longer in MI segments compared to normal and peri-MI segments at the early (440 ± 40 vs. 329 ± 40 and 332 ± 36 ms, respectively; p = 0.0002) and late post-MI time-points (442 ± 63 vs. 321 ± 40, 355 ± 61, respectively; p = 0.012; Table 3, Figure 4).

#### Analysis by time point

TTP in normal segments did not change significantly from pre-MI to early to late post MI (300 ± 46 ms, 329 ± 40 ms, 321 ± 40 ms, p = 0.24, 0.39 and 0.74, respectively). TTP in peri-MI segments did not change significantly from pre-MI to early to late post MI (300 ± 46 ms, 332 ± 36 ms, 355 ± 61 ms, p = 0.07, 0.28 and 0.43, respectively). TTP in MI segments increased significantly from pre-MI to early and late post MI (300 ± 46 ms, 440 ± 40 ms, 442 ± 63 ms, p = 0.0002). There was no significant increase in TTP between early and late post MI (440 ± 40, 442 ± 63, p = 0.92; Table 2, Figure 4).

#### Standard deviation (Dyssynchrony Index)

The SD16 [19,20] significantly increased from 28 ± 7 ms at baseline to 50 ± 10 ms at early (p = 0.012) and 54 ± 19 ms (p = 0.004) at late post-MI time-points, respectively. However, there was no significant difference in SD16 between early and late post-MI time-points (p = 0.56), suggesting that significant dyssynchrony occurs early after MI without significant worsening in the ensuing weeks (Table 2).

To specifically address whether longer time-points would alter our observations, we performed similar analysis on 2 animals at 135 days after MI. Although statistical analysis is not feasible at this sample size, the TTP results in the MI segments between early, late and 135-day studies were similar. Our findings were: Animal # 1) 472 ms (early), 460 ms (30 days) and 483 ms (135 days); Animal # 2) 492 ms (early), 512 ms (30 days) and 450 ms (135 days). These 2 case examples appear to support our earlier observations concerning the lack of significant additional dyssynchrony between 1 and 4 weeks of post-MI.

We found no relationship between regional strain and dyssynchrony when considering normal segments (R^2 ^= 0.01, p = 0.14), peri-MI (R^2 ^= 0.02, p = 0.3) and MI segments (R^2 ^= 0.02, p = 0.4).

Inter-observer reproducibility of TTP was tested in 63 random segments at late post-MI. Mean TTP was 304 ± 48 ms and 313 ± 55 ms for observer 1 and 2, respectively, with a mean difference of 9 ms (coefficient of variation 3%).

## Discussion

In a closed-chest porcine model, mechanical dyssynchrony, as evidenced by the standard deviation in TTP, occurs early after MI and does not significantly worsen in the near term. Dyssynchrony originates primarily from delayed electrical and mechanical activation of the infarcted region.

Myocardial infarction and its common consequence, heart failure, present a significant health problem in the United States and the world [22]. Despite strong clinical gains from CRT in the overall heart failure population, results in ischemic CM have been underwhelming [23]. Areas of infarction have delayed mechanical activation due to local conduction abnormalities, delays in electro-mechanical coupling, and myocardial dysfunction. However, the mechanical relationship between infarct areas and peri-infarct myocardium is unclear [24]. Moreover, the mechanical behavior of the peri-infarct zone with respect to dyssynchrony is unclear. To address this knowledge gap we studied regional mechanics early and late post-MI in an extensively characterized porcine model of MI [15,24,25]. We selected a closed-chest approach to avoid the confounding influence of sternotomy and pericardiectomy that are known to affect myocardial mechanics. This model exhibited all the classic features of morphologic and functional remodeling seen in clinical and experimental MI. Additionally, using high resolution ex vivo CMR in this animal model, Ashikaga et al [26] have demonstrated a complex 3D structure of the scar: they found a thin rim of viable myocardium on the endocardial aspects of the scar (endocardial border-zone) and islands of viable myocardium within transmural-appearing scar that would result in fragmented electrograms and delayed activation of the infarcted region (by endocardial mapping).

Our model allows us to reliably characterize the distribution and extent of infarct by LGE. Similarly, we were able to evaluate the time course of changes in regional contractility and dyssynchrony following MI using CMR tagging. Unlike echocardiography, tagged CMR allows evaluation of myocardial strain at high spatial resolution and reproducibility. Strain, which evaluates regional myocardial deformation, is more reflective of myocardial mechanics than displacement mapping using parameters such as tissue velocity which are prone to artifacts from translational motion and tethering [27]. These artifacts may lead to high variability in tissue velocity-based indices of dyssynchrony [28]. This advantage of strain over velocity mapping is more pronounced in regional pathologies such as myocardial infarction [27]. The dyssynchrony index used in this study has been previously validated in CMR based studies of dyssynchrony [19,20]. It offers the best snapshot of the mechanical behavior of individual segments relative to the entire heart provides a numerically expression for the temporal dispersion in mechanical activity in the heart. One potential advantage of CMR based zHARP assessment of dyssynchrony is that the multiple peaks, often noted in echo-based tissue velocity traces, were not observed in this study. However, this observation may require a wider population to be confirmed.

Our study demonstrates significant mechanical dyssynchrony within days of an MI, which is in concordance with previously published work using echocardiography in clinical populations [11]. Echocardiographic tissue velocity-based dyssynchrony indices suggest a standard deviation of time to peak displacement of approximately 30 ms represents significant mechanical dyssynchrony [28,29]. Although we cannot directly extrapolate these echo-based results, it is noteworthy that our index of global dyssynchrony (SD16) was significantly abnormal at 1 week post-MI (50 ms). Our data show that dyssynchrony occurs early and provide insights into why echo-based evaluation of dyssynchrony days after MI was highly predictive of long term outcomes [8,10]. However, since different imaging methodologies and indices were used in our study versus the previous echocardiography-based studies, wider, direct comparisons between our data and previous echo-based data are difficult. Furthermore, the subjects are not exactly the same, since patients may have more extensive disease and larger MIs, including acute on chronic ischemia and multi-vessel disease, compared to the well-circumscribed, relatively small apical MI in our model. We did not evaluate longitudinal displacement as done by Zhang et al [8] and decided not to use radial strain, as done by Mollema et al. [10], since we were unable to obtain adequate quality εR tracings by CMR. Recent data questioning the reliability and therefore usefulness of echo-based dyssynchrony indices also need to be considered when comparing our results to these previous publications [28]

Another important finding of our study is that delayed mechanical activation is not linked to reduced regional function per se. Regional contractility and conduction velocity were lowest in MI segments and intermediate in peri-MI segments, unlike the study by Klemm et al. [30] in patients with ischemic cardiomyopathy, which found viability and increased CV in areas with slow wall motion. We did not find mechanical delays in the peri-MI zones despite reduced regional function at 1 week post MI. Whether this is due to mechanical tethering of the peri-MI segments to the normal segment or a true lack of regional dyssynchrony could not be assessed by our study.

Conduction velocity was significantly lower in the infarct and infarct border-zone (IBZ) when compared to the remote myocardium. The infarct was the latest activated region because of very slow conduction, indicating that reduction in wave propagation velocity is the most important contributor to the time delay in regional contraction that we observed. Impulse propagation in the heart is dependent on active membrane properties determined by the ion channel composition, cell size, gap junction function and distribution [31]. Previous work [32] has demonstrated that surviving myocytes in the healed IBZ have normal resting membrane potential and normal action potential morphologies. However, CV can be reduced in the IBZ due to distortion of myocyte alignment (non-uniform anisotropy), interstitial fibrosis, and/or gap junction remodeling [32,33]. This may explain the reduction in CV without significant change in TTP in the IBZ. Alterations in Ca^2+^handling and Ca^2+ ^transients that have been previously reported in infarct border-zone myocytes [34], in combination with non-uniform anisotropy in the IBZ could manifest as reduced peak systolic strain.

Another possible explanation for lack of a relationship between delayed mechanical activation and reduced regional function is infarct expansion, although we did not see an increase in the number of delayed-enhancement segments late post-MI, suggesting this was not a dominant mechanism in our study. Lastly, differences may be due to segment definition, compared to previous studies: a peri-MI segment in our study was a segment adjacent to an MI segment (Figure 1) and was not a partial thickness MI. We used this definition as in our model of a well-circumscribed MI, there were few if any segments with partial thickness LGE.

There are several clinical implications of our findings for CRT in patients with ischemic CM. Our data indicate that *1) *delayed electrical and mechanical activation of the infarct is the main cause of dyssynchrony; *2) *despite adverse remodeling of the left ventricle post-MI, further worsening of mechanical dyssynchrony does not occur. Hence, assessment of dyssynchrony one week post-MI or before discharge from the hospital, should be adequate to assess the consequences of MI on mechanical synchrony. The one week time point was chosen for logistical reasons in this animal study. However, based on the evolution of myocardial infarction, imaging any time in the first week post-MI should suffice. While the relationship between scar burden and lack of response to CRT in patients with ischemic CM has been reported before [35,36], the underlying mechanism has not been completely elucidated. Based on our results, the current standard practice of pacing the lateral wall is unlikely to substantially change global dyssynchrony unless the infarct is in the paced region. Additionally, pacing a normal region may in fact worsen cardiac mechanics in ischemic CM by bypassing the His-Purkinje system and relying on cell-cell electrical propagation. Simply placing the LV lead in an infarcted territory may also not be the best option [6] since these segments would be unable to respond mechanically because of inadequate viable myocardium. Hence, despite the presence of mechanical dyssynchrony, patients with transmural infarcts may not respond to traditional CRT post-MI when the late activated region corresponds to the infarct. Also, beta blockers that reduce adverse remodeling and improve mechanical dyssynchrony in non-ischemic cardiomyopathy may not be effective in reducing dyssynchrony post-MI, because dyssynchrony [37] was caused by massive loss of myocytes in the infarcted region and was not the result of adverse remodeling. Based on our results, this type of dyssynchrony would be most amenable to strategies that promote regeneration of viable myocardium and improvement of conduction in the infarcted region [38].

## Limitations of the study

The sample size is relatively small. However, power calculations and our results suggest that it is adequate for this analysis. Larger MIs resulted in significant animal mortality so we limited the size of the MI to approximately 20%. Hence, we are unable to assess the relationship between infarct size and dyssynchrony. Additionally, we did not study changes in regional mechanics beyond 4 weeks; a longer term study is logistically challenging and prohibitively expensive. Since this is not an intervention study we cannot assess the response of ischemic CM to CRT or medications like beta blockers. Furthermore, due to poor radial strain quality we were unable to examine the relative value of eR and eC. However, the low feasibility of eR suggests it may not be as useful in this model. Lastly, due to logistic difficulties we were unable to perform concomitant echocardiography evaluation of dyssynchrony in this study that would have allowed better comparisons to published echo-based data. Conduction velocity measurements using EAM are sensitive to mapping density as well as underlying myocardial architecture (uniform anisotropy), with longitudinal CV being significantly greater than transverse CV in normal myocardium. This could have resulted in the large variation in CV observed in normal myocardium. Lastly, we did not isolate myocytes for cellular studies which could have provided insights into the electrical and mechanical changes that we observed.

## Conclusions

Mechanical dyssynchrony occurs early after acute MI with non-significant changes in the near term. Delayed mechanical activation is noted primarily in the MI segments. Further imaging and cellular studies are needed to investigate dyssynchrony and the effects of interventions such as beta blockers, stem cell therapy and CRT in ischemic cardiomyopathy.

## List of abbreviations used

**CRT: **cardiac resynchronization therapy; **LV: **left ventricle; **CM: **cardiomyopathy; **MI: **myocardial infarction; **CMR: **cardiovascular magnetic resonance, **zHARP: **z-encoding harmonic phase imaging; **EAM: **Electroanatomical Mapping; **eC: **Circumferential Strain; **eR: **Radial strain; **TTP: **Time to peak strain; **EF: **Ejection Fraction; **IBZ: **Infarct border-zone; **CV: **Conduction velocity.

## Competing interests

Dr. Calkins receives research support from Medtronic, St Jude Medical, and Boston Scientific and serves as a consultant for Medtronic. All other authors have reported that they have no relationships to disclose.

## Authors' contributions

KZA: made substantial contributions to acquisition and analysis of data and has been involved in drafting and revising the manuscript. MST: made substantial contributions to analysis and interpretation of data and has been involved in drafting and revising the manuscript. TS: made substantial contributions to conception and design and acquisition of data and has been involved in revising the manuscript. SS: made substantial contributions to analysis and interpretation of data and has been involved in revising the manuscript. EVP: made substantial contributions to acquisition and analysis of data. AY: made substantial contributions to acquisition of data. HA: made substantial contributions to data analysis and has been involved in drafting the manuscript. VLD: made substantial contributions to analysis and interpretation of data and has been involved in revising the manuscript. HC: made substantial contributions to data acquisition and interpretation. JLP: made substantial contributions to conception, design and interpretation of data and has been involved in revising the manuscript. TPA: made substantial contributions to conception, design and analysis and interpretation of data and has been involved in drafting and revising the manuscript. MRA: made substantial contributions to conception, design, acquisition and interpretation of data and has been involved in drafting and revising the manuscript. All authors read and approved the final manuscript.

## Supplementary Material

Additional file 1 **Supplemental Material and Methods**. The file contains detailed descriptions of the cine, tagging, and delayed enhancement imaging protocols and image analyses performed in this study. Click here for file

Additional file 2 **Graphical description of infarct, peri-infarct and normal segments**. The figure shows 1) infarct segments which were defined as those with > 25% delayed enhancement and < 10% strain, 2) peri-MI segments were defined as those immediately adjacent to an MI segment in the 3-dimensional space, and 3) the remaining segments which were considered as normal segments. Click here for file
