# Supplementary material for: Assessment of distribution and evolution of Mechanical dyssynchrony in a porcine model of myocardial infarction by cardiovascular magnetic resonance
Source: J Cardiovasc Magn Reson. 2012 Jan 6;14(1):1. doi: 10.1186/1532-429X-14-1 (PMC3268109; doi:10.1186/1532-429X-14-1)
Supplement: Additional file 1 — Supplemental Material and Methods. The file contains detailed descriptions of the cine, tagging, and delayed enhancement imaging protocols and image analyses performed in this study. [file 1532-429X-14-1-S1.DOC]

**Supplemental Material and Methods**

for the following article

**TITLE**: **Assessment of Distribution and Evolution of Mechanical Dyssynchrony in a Porcine Model of Myocardial Infarction by Cardiac Magnetic Resonance**

Khaled Z. Abd-Elmoniem, PhD^1*^, Miguel Santaularia Tomas, MD^2,3*^, Tetsuo Sasano, MD^2*^, Sahar Soleimanifard, MSE^4^, Evert-Jan P. Vonken, MD, PhD^2^, Amr Youssef, MD^2^, Harsh Agarwal, PhD^4^, Veronica L. Dimaano, MD^2^, Hugh Calkins, MD^2^, Matthias Stuber, PhD^5^, Jerry L. Prince, PhD^4^, Theodore P. Abraham, MD^2^, M. Roselle Abraham, MD^2§^

_____________________________________________________________________

**Creation of Myocardial Infarction**: Briefly, anesthesia was induced using a weight-based cocktail (Telazol 1.5 mg/kg, Ketamine 1.5 mg/kg and Xylazine 1.5 mg/kg) and maintained with 1.5% to 2% inhalational Isoflurane. All animals were maintained on mechanical ventilation. Under fluoroscopic guidance, using an over-the-wire technique, an angioplasty balloon was advanced to a location just distal to the second diagonal branch of the left anterior descending coronary artery and inflated to occlude the artery for 150 minutes. Prophylactic intravenous Lidocaine was administered and episodes of ventricular fibrillation were treated with electrical defibrillation. Post-procedure electrocardiography (ECG) showed no prolongation of QRS duration or bundle branch block. The animal was recovered and returned to the vivarium. All animals received postoperative pain control consisting of weight-based buprenorphine and ketorolac. Surviving animals were monitored daily for the period of the study. Imaging studies were performed pre- MI (baseline), at one week post-MI, and at approximately 4 weeks post-MI. These particular time points were selected for pathologic, clinical and logistic reasons. Pathologically, infarcts are more distinct at 5-7 days after the event and infarct healing (fibrosis) is considered complete in a month [1]. Clinically, various parameters of heart size and function (including mechanical dyssynchrony), assessed days after an infarct predict long-term clinical outcomes [2-4]. Furthermore, it is recommended that decisions regarding device therapy in the post-MI patient be made at least 3 weeks after the infarct [5]. Device therapy applied based on LV assessment at earlier time points has not proven to be beneficial [6].

**Cardiac Magnetic Resonance**: Studies were performed with a clinical Philips 3.0T Achieva MRI scanner (Philips Healthcare, Best, NL) equipped with a six-channel cardiac phased array surface coil. Channels were distributed equally between the anterior and posterior side of the chest. Four ECG leads were placed on the pig’s chest for triggering the pulse sequence at the R-wave of the ECG. The animals were pre-medicated intramuscularly with droperidol and fentanyl citrate (Innovar 0.1 ml/kg), intubated, anesthetized with IV sodium pentobarbital (20-25 mg/kg), mechanically ventilated, and positioned head first and supine in the scanner. Animals were mechanically ventilated, anaesthetized and paralyzed (Vecuronium 0.1mg/kg) for the duration of imaging. All pigs underwent cine, tagged and delayed Gadolinium contrast-enhanced (DE) MR imaging to analyze global, and regional left ventricular function, and MI location, respectively. Ventilation was suspended during all image acquisition (except for DE images).

**Cine Images:** After scout images were obtained, 9 to 12 contiguous short-axis slices were prescribed to cover the entire LV. Cine images were acquired using a steady state free precession pulse sequence: repetition time (TR) 3.4 ms, echo time (TE) 1.69 ms, flip angle 40°, average in-plane resolution 1.59x1.34 mm^2^, slice thickness 8 mm, 0 mm slice gap, and temporal resolution 20 ms. The average breath hold was 14-17 s, and 39 cardiac cycles were used to complete a cine sequence. A simple arrhythmia rejection scheme was implemented in which dissimilar heartbeats or heartbeats with short RR interval or premature ventricular contractions were manually discarded from the selective average. There were no arrhythmias or premature beats; hence no cycles were discarded in our sample.

**Contrast Enhanced CMR :** DE images were acquired in early and late post-MI scans 7 to 10 minutes after a total injection of 0.2 mmol/kg gadodiamide (Omniscan, GE Healthcare Technologies) with a free-breathing, navigator-guided 3D phase sensitive inversion recovery fast gradient-echo pulse sequence [7]. Imaging parameters were as follows: TR 4.6 ms, TE 2.2 ms, flip angle 15°, average in-plane resolution 1.7x1.9 mm^2^, 3 mm slice thickness, 0 mm slice gap, average inversion time (TI) 320 ms (adjusted to null the signal of normal myocardium), 1 excitation, 2 R-R interval imaging, 675 ms time delay after the R wave, and 25 views per segment. These parameters yielded an image acquisition window of 136 ms.

**Tagged CMR:** For tagged CMR imaging, 9 to 12 tagged equally spaced short-axis sections were acquired to cover the entire left ventricle from the base to the apex in the same short-axis locations as the cine images. The tagging pulse sequence consisted of slice-following selective radiofrequency pulses separated by spatial modulation of magnetization-encoding with z-encoding gradients (zHARP; z harmonic phase imaging) to achieve tag spacing of 7 mm [8-10]. In addition to encoding both in-plane and through-plane displacements of an imaged slice, zHARP tagging combines tools for enhancing the accuracy and reproducibility of strain measurement. It utilizes complementary tagging with variable radio frequency flip angle to enhance myocardial tagging signal-to-noise-ratio at later heart phases [11], slice-following [12] to track and image the same anatomical slice of the myocardium throughout the cardiac cycle, and harmonic peak combination [13] for field inhomogeneity artifact correction. Images were acquired during 30-50 seconds breath holds requiring 3 breath holds per slice. The scans were performed using vector electrocardiogram triggered spoiled gradient-echo pulse sequence with segmented k-space spiral acquisition with spectral-spatial excitation [14]. The parameters for tagged MR imaging were as follows: TR 20 ms, TE 2.5 ms, flip angle 15°, slice thickness 8 mm; field of view 320x320 mm^2^; matrix size 256x256, 12 spiral interleaves, and acquisition window 12 ms. Cardiac shimming was performed before each sequence to minimize artifacts due to field inhomogeneities [15, 16].

**Image Analysis**

LV volumes and global LV function were analyzed off-line on a personal computer using a custom-built software tool developed using Matlab® ver. 7.6 (Mathworks, Natick, MA). Global function was assessed by calculating ejection fraction (EF; difference between end-diastolic and end-systolic volumes normalized to end-diastolic volume, expressed as a percentage). Technical details of CMR tagging and HARP analysis have been previously described. Image processing was performed off-line on a personal computer using a customized HARP analysis program written in MATLAB. The software prompts the operator to identify a center point in a mid-ventricular short axis view followed by manual segmentation of the endocardial and epicardial contours at each ventricular level (base, mid and apical). Papillary muscles were located in cine images, matched with the HARP data and used as a reference point to divide the LV into basal, mid and apical sections. End-systole was defined as the time point at which minimal left ventricular volume was observed and end-diastole as the time point of maximal ventricular volume. Special care was taken to ensure that outer and inner contour margins were located within the epicardial and endocardial borders to optimize tracking of all endocardial segments; papillary muscles were excluded. For delineation of myocardial segments, the program prompts the operator to identify the two points of insertion of the right ventricular free wall into the septum. Based on these anatomical landmarks, the program automatically divides each short axis view into 6 segments of equal radial length. This process is applied at the basal and mid levels. The program is modified at the apical level to yield 4 apical segments such that the heart is finally divided into the standard 16 segment model [17]. DE image analysis was performed using a customized MATLAB program. Prior to analysis of contrast images, each HARP slice was spatially matched to its corresponding contrast image slice using a function in the custom HARP analysis software. Subsequently, a second segmentation was performed using to yield a 16-segment model of the contrast images such that the segments in the HARP and contrast analysis were spatially matched. Finally, DE images were used to determine scar size (expressed as percentage of total LV myocardial volume). In our study, DE image slices showed a clear delineation between normal and infarcted myocardium, and all infarcts were transmural thus no transmurality analysis was done. An experienced cardiac imager manually outlined endocardial and epicardial borders on the short-axis DE images. To avoid partial volume effects, the most apical and the most basal slices in which the wall thickness was < 50% of the wall thickness at the same level (outflow tract location) were excluded from analysis. Infarct (MI) and peri-MI segments were defined based on tagged MRI and DE information. Infarct segments were defined as a segment with >25 % delayed enhancement and <10 % strain. This definition minimized the chance of mis-classification due to any slice mis-registration. Peri- MI segments were defined as those immediately adjacent to an MI segment, sharing a border in the 3-dimensional space, i.e. an in-plane segment adjacent to the MI segment and a segment in the z- axis adjacent to the MI (mid segment relative to an apical MI segment) were both defined as peri-MI (Supplemental Figure 1). The remaining segments were considered as normal segments. These definitions provided the highest intra- and inter- observer reproducibility. We did examine alternative options for segment definition based on signal intensity such as full width at half maximum method and 2 standard deviation but these techniques resulted in significant variability in MI segment sizes. Segmental circumferential (eC) and radial (eR) strains were calculated over multiple cardiac phases. By convention, systolic eC is denoted by a negative value indicating shortening of the distance between 2 material points in the circumferential direction. Similarly, systolic eR is denoted by a positive value indicating lengthening of the distance between 2 material points in the radial direction. Time to peak strain was determined for each segment by measuring the time from the start of the cardiac cycle to the maximal systolic amplitude of the strain curve for that segment. Mechanical dyssynchrony was calculated at baseline, early and late post MI, by means of a dyssynchrony index [18-20]. Dyssynchrony index was calculated as the standard deviation of time to peak eC (TTP) for 16 segments per animal. This index has been previously used to assess dyssynchrony in experimental and clinical studies [21-24]. The primary comparisons were made between infarct, peri-infarct and normal segments at three time-points, namely, pre-MI (baseline), early (7 days) and late (30-40 days) post-MI.

**Electroanatomical Mapping (EAM).**

EAM was performed using the CARTO system (CARTO XP, Biosense-Webster Inc.) in all pigs within 24hrs of MR imaging and prior to sacrifice. The LV and RV endocardium was fully mapped (to achieve a fill threshold <15mm and <20mm, respectively) during sinus rhythm. For voltage mapping, a bipolar voltage (BV) <0.5mV was defined as infarct scar according to previously established criteria [25]. Isochrone maps were constructed and conduction velocity was calculated in the infarct, border-zone and remote myocardium.

References:

1. Burke AP, Virmani R: Pathophysiology of acute myocardial infarction. *Med Clin North Am* 2007, 91:553-572; ix.

2. Ko JS, Jeong MH, Lee MG, Lee SE, Kang WY, Kim SH, Park KH, Sim DS, Yoon NS, Yoon HJ, et al: Left Ventricular Dyssynchrony After Acute Myocardial Infarction is a Powerful Indicator of Left Ventricular Remodeling. *Korean Circ J* 2009, 39:236-242.

3. Mollema SA, Liem SS, Suffoletto MS, Bleeker GB, van der Hoeven BL, van de Veire NR, Boersma E, Holman ER, van der Wall EE, Schalij MJ, et al: Left ventricular dyssynchrony acutely after myocardial infarction predicts left ventricular remodeling. *J Am Coll Cardiol* 2007, 50:1532-1540.

4. Temporelli PL, Giannuzzi P, Nicolosi GL, Latini R, Franzosi MG, Gentile F, Tavazzi L, Maggioni AP: Doppler-derived mitral deceleration time as a strong prognostic marker of left ventricular remodeling and survival after acute myocardial infarction: results of the GISSI-3 echo substudy. *J Am Coll Cardiol* 2004, 43:1646-1653.

5. Epstein AE, DiMarco JP, Ellenbogen KA, Estes NA, 3rd, Freedman RA, Gettes LS, Gillinov AM, Gregoratos G, Hammill SC, Hayes DL, et al: ACC/AHA/HRS 2008 Guidelines for Device-Based Therapy of Cardiac Rhythm Abnormalities: a report of the American College of Cardiology/American Heart Association Task Force on Practice Guidelines (Writing Committee to Revise the ACC/AHA/NASPE 2002 Guideline Update for Implantation of Cardiac Pacemakers and Antiarrhythmia Devices) developed in collaboration with the American Association for Thoracic Surgery and Society of Thoracic Surgeons. *J Am Coll Cardiol* 2008, 51:e1-62.

6. Hohnloser SH, Kuck KH, Dorian P, Roberts RS, Hampton JR, Hatala R, Fain E, Gent M, Connolly SJ: Prophylactic use of an implantable cardioverter-defibrillator after acute myocardial infarction. *N Engl J Med* 2004, 351:2481-2488.

7. Kellman P, Arai AE, McVeigh ER, Aletras AH: Phase-sensitive inversion recovery for detecting myocardial infarction using gadolinium-delayed hyperenhancement. *Magn Reson Med* 2002, 47:372-383.

8. Abd-Elmoniem KZ, Osman NF, Prince JL, Stuber M: Three-dimensional magnetic resonance myocardial motion tracking from a single image plane. *Magn Reson Med* 2007, 58:92-102.

9. Abd-Elmoniem KZ, Sampath S, Osman NF, Prince JL: Real-time monitoring of cardiac regional function using fastHARP MRI and region-of-interest reconstruction. *IEEE Trans Biomed Eng* 2007, 54:1650-1656.

10. Abd-Elmoniem KZ, Stuber M, Prince JL: Direct three-dimensional myocardial strain tensor quantification and tracking using zHARP. *Med Image Anal* 2008, 12:778-786.

11. Fischer SE, McKinnon GC, Maier SE, Boesiger P: Improved myocardial tagging contrast. *Magn Reson Med* 1993, 30:191-200.

12. Fischer SE, McKinnon GC, Scheidegger MB, Prins W, Meier D, Boesiger P: True myocardial motion tracking. *Magn Reson Med* 1994, 31:401-413.

13. Ryf S, Tsao J, Schwitter J, Stuessi A, Boesiger P: Peak-combination HARP: a method to correct for phase errors in HARP. *J Magn Reson Imaging* 2004, 20:874-880.

14. Meyer CH, Pauly JM, Macovski A, Nishimura DG: Simultaneous spatial and spectral selective excitation. *Magn Reson Med* 1990, 15:287-304.

15. Kubach MR, Bornstedt A, Hombach V, Merkle N, Schar M, Spiess J, Nienhaus GU, Rasche V: Cardiac phase-specific shimming (CPSS) for SSFP MR cine imaging at 3 T. *Phys Med Biol* 2009, 54:N467-478.

16. Schar M, Kozerke S, Fischer SE, Boesiger P: Cardiac SSFP imaging at 3 Tesla. *Magn Reson Med* 2004, 51:799-806.

17. Cerqueira MD, Weissman NJ, Dilsizian V, Jacobs AK, Kaul S, Laskey WK, Pennell DJ, Rumberger JA, Ryan T, Verani MS: Standardized myocardial segmentation and nomenclature for tomographic imaging of the heart: a statement for healthcare professionals from the Cardiac Imaging Committee of the Council on Clinical Cardiology of the American Heart Association. *Circulation* 2002, 105:539-542.

18. Miyazaki C, Lin G, Powell BD, Espinosa RE, Bruce CJ, Miller FA, Jr., Karon BL, Rea RF, Hayes DL, Oh JK: Strain dyssynchrony index correlates with improvement in left ventricular volume after cardiac resynchronization therapy better than tissue velocity dyssynchrony indexes. *Circ Cardiovasc Imaging* 2008, 1:14-22.

19. Yu CM, Bax JJ, Monaghan M, Nihoyannopoulos P: Echocardiographic evaluation of cardiac dyssynchrony for predicting a favourable response to cardiac resynchronisation therapy. *Heart* 2004, 90 Suppl 6:vi17-22.

20. Yu CM, Fung JW, Zhang Q, Chan CK, Chan YS, Lin H, Kum LC, Kong SL, Zhang Y, Sanderson JE: Tissue Doppler imaging is superior to strain rate imaging and postsystolic shortening on the prediction of reverse remodeling in both ischemic and nonischemic heart failure after cardiac resynchronization therapy. *Circulation* 2004, 110:66-73.

21. Aiba T, Hesketh GG, Barth AS, Liu T, Daya S, Chakir K, Dimaano VL, Abraham TP, O'Rourke B, Akar FG, et al: Electrophysiological consequences of dyssynchronous heart failure and its restoration by resynchronization therapy. *Circulation* 2009, 119:1220-1230.

22. Barth AS, Aiba T, Halperin V, DiSilvestre D, Chakir K, Colantuoni C, Tunin RS, Dimaano VL, Yu W, Abraham TP, et al: Cardiac resynchronization therapy corrects dyssynchrony-induced regional gene expression changes on a genomic level. *Circ Cardiovasc Genet* 2009, 2:371-378.

23. Chakir K, Daya SK, Tunin RS, Helm RH, Byrne MJ, Dimaano VL, Lardo AC, Abraham TP, Tomaselli GF, Kass DA: Reversal of global apoptosis and regional stress kinase activation by cardiac resynchronization. *Circulation* 2008, 117:1369-1377.

24. Miyazaki C, Redfield MM, Powell BD, Lin GM, Herges RM, Hodge DO, Olson LJ, Hayes DL, Espinosa RE, Rea RF, et al: Dyssynchrony indices to predict response to cardiac resynchronization therapy: a comprehensive prospective single-center study. *Circ Heart Fail* 2010, 3:565-573.

25. Marchlinski FE, Callans DJ, Gottlieb CD, Zado E: Linear ablation lesions for control of unmappable ventricular tachycardia in patients with ischemic and nonischemic cardiomyopathy. *Circulation* 2000, 101:1288-1296.

**Figure 1:** Infarct segments were defined as those with >25 % delayed enhancement and <10 %

strain. Peri-MI segments were defined as those immediately adjacent to an MI segment in the 3-

dimensional space. The remaining segments were considered as normal segments.
