# Supplementary figures and images for: Assessment of distribution and evolution of Mechanical dyssynchrony in a porcine model of myocardial infarction by cardiovascular magnetic resonance
Source: J Cardiovasc Magn Reson. 2012 Jan 6;14(1):1. doi: 10.1186/1532-429X-14-1 (PMC3268109; doi:10.1186/1532-429X-14-1)

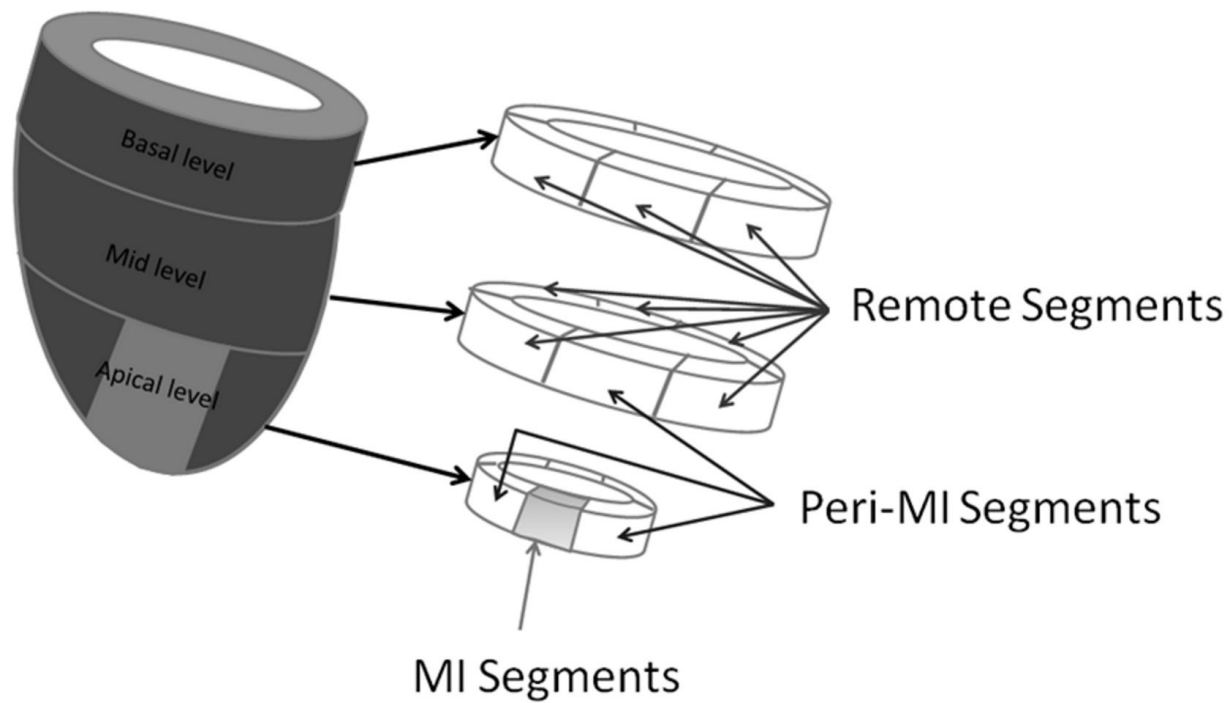

Supplement: Additional file 2 — Graphical description of infarct, peri-infarct and normal segments. The figure shows 1) infarct segments which were defined as those with > 25% delayed enhancement and < 10% strain, 2) peri-MI segments were defined as those immediately adjacent to an MI segment in the 3-dimensional space, and 3) the remaining segments which were considered as normal segments. [file 1532-429X-14-1-S2.PDF]
